# Supplementary material for: Are carbon emissions trading and green financial instruments synergistic? -Comprehensive quantitative research based on content analysis
Source: PLoS One. 2024 Mar 7;19(3):e0298601. doi: 10.1371/journal.pone.0298601 (PMC10919601; doi:10.1371/journal.pone.0298601)
Supplement: S1 Data — (ZIP) [file pone.0298601.s001.zip › Data/地级市Z_F工作报告.zip!/░▓╗╒/╗┤▒▒╩╨/└┤╫╘╣1⁄2╓┌║┼í╛┬φ┐╦╩2╛▌═°í┐_2013.docx]

各位代表：
　　我受市第十四届人大常委会的委托，向大会报告本届常委会的主要工作，请予审议，并请列席会议的同志提出宝贵意见。
　　 ****一、迎难而上、转型攻坚的奋进历程****

    2012年是我市发展历程中极不平凡的一年。面对宏观经济下行,煤炭产业遭遇十年来最严重挑战等复杂严峻形势,在中共淮北市 委的坚强领导下,市政府团结带领全市人民,深入贯彻落实科学发展观,牢牢把握稳中求进、好中求快的工作总基调,扎实开展“转型发展攻坚年”活动,着力稳增长调结构、打基础增后劲、惠民生促和谐,地区生产总值、规模以上工业增加值、固定资产投资、城镇居民人均可支配收入等主要经济指标增幅在全省位次大幅前移,创五年来最好水平,较好地完成了市十四届 人大五次会议确定的目标任务。
　　预计全年完成地区生产总值640亿元,比上年增长13.2%;财政收入101亿元,增长11.7%,其中地方财政收入52亿元,增长36.5%;固定资产投资570亿元,增长27%;城镇居民人均可支配收入20650元,增长15.5%,农民人均纯收入7480元,增长18.5%;城镇登记失业率控制在4.1%以内;节能减排完成省政府下达的目标任务。全市经济社会发 展呈现稳中有进、结构优化、转型加速、活力增强、民生改善、社会和谐的良好态势。
　　一年来,我们主要做了以下工作:(一)稳增长调结构,经济发展的质量和效益显著提高工业经济加速发展。坚持把工业强市作为稳增长的主攻方向,出台促进经济稳定增长40条意见,扎实开展百名干部驻点帮 扶百家企业活动,采取“一企一策”缓解企业经营困难,全年落实扶企资金4.3亿元,发放中期票据、助保金贷款42.7亿元。实施“万千百十”工程,推动企业上规模上台阶,全年新增规模以上工业企业120家,总数达620家。年销售收入超亿元企业300家,超10亿元企业20家,淮海实业首次跨过百亿元台阶,全市百亿元企业增至3家。
　　淮北矿业和皖北煤电在中国企业500强的排名,分别比上年提升16和24个位次。预计全年实现规模以上工业增加值488亿元,居全省第4位,前移2个位次。
　　有效投入持续扩大。坚持把扩投入作为稳增长的关键举措,创新项目谋划机制,充实完善市级项目库,全年谋划重大项目267个,总投资1440亿元。健全项目推进机制和市级领导联系督查重点项目制度,325个重点项目完成投 资380亿元,210个省“861”行动计划项目完成投资102.6亿元,155个央企、民企、皖粤合作项目完成投资160亿元。安徽矿机、百特电机等重点项目建成投产,临涣煤焦化综合利用二期开工建设,虎山电厂、平山电厂一期顺利推进。淮海建材大市场建成运营,红星美凯龙、国购汽车文化园、口子文化博览园等商贸、旅游项目加快建设。
　　消费需求不断拓展。坚持把促消费作为稳增长的持久动力，加强市场流通体系建设，扩大社区商业、家政等便民消费，发展旅游休闲、文化娱乐等服务消费。成功举办首届食品工业博览会、第七届煤矿机械博览会和第三届塔山石榴文化节，认真落实家电下乡、节能产品惠民、农产品产销对接等促进消费政策。预计全年实现社会消费品零售总额171.3亿元，增长15.8%，进出口总额3.4亿美元，增长41.6%。
　　采取多种措施稳定房地产市场，全年完成投资101.7亿元，增长52.8%，增速居全省首位，新开工面积426万平方米，竣工面积25万平方米。
　　结构调整扎实推进。坚持把转方式、调结构作为经济工作的主线，大力实施“转型发展攻坚年”活动，转型升级取得重要进展。非煤产业产值占全市工业总产值比重达65%，比上年提高7.2个百分点。积极培育战略性新兴产业，实现产值220亿元，增幅居全省前列。县区经济发展强劲，县区生产总值、规模工业增加值占全市的比重分别达62.2%和42.7%，同比提高4.2和5.3个百分点。非公经济增加值占全市生产总值比重达41%；服务业增加值159亿元，占全市生产总值比重达24.8%。
　　（二）强基础促统筹，“三农”工作全面加强现代农业加快发展。认真落实强农惠农富农政策，全面提升农业综合生产能力，粮食总产达119万吨，成功实现“七连增、九连丰”。特色产业基地和现代农业示范园加快发展，“塔山石榴”成国家地理标志产品。全年新增土地流转面积10万亩，市级以上龙头企业增至122家，农村经济合作组织发展到838家。完成农田水利基本建设投资1.8亿元，新增和改善灌溉面积31.2万亩、除涝面积18.4万亩。
　　城乡一体化加快推进。市财政安排专项资金1亿元，重点支持24个城乡一体化和新农村项目建设，带动社会投资近20亿元。通过“农民集中建房+政府补贴”方式，积极开展土地整治整村推进工作，建设农村新型社区，开工建设新农村示范点7个、居民集中居住区14个。全面启动美好乡村建设，加快编制37个中心村规划，榴园村等成为全省亮点。在全省较早推进户籍制度改革，鼓励农民变市民。积极落实扩权强镇各项政策，濉溪县临涣镇获批为第三批全国发展改革试点镇。
　　（三）抓改革促创新，发展活力日益彰显着眼于完善体制机制，破解发展难题，扎实推进重点领域和关键环节改革。事业单位清理规范工作基本完成，文化体制改革不断深化，医药卫生体制改革加快推进。企业统计联网直报改革顺利完成，城乡收入一体化统计改革正式启动。蚌埠海关驻淮北工作组通关运行，“营改增”试点稳步实施，投融资体制改革、金融创新取得新成效。在全省率先启动农村土地承包经营权抵押贷款试点。市交通投资公司挂牌。交通银行、招商银行分支机构在淮设立。网络金融超市建成运营。不断拓宽融资渠道，争取国家开行等金融机构融资额度134亿元，到位67.8亿元。市建投公司18亿元企业债券成功发行，6年期以上融资年利息不超过7%。4家企业实现直接融资，共筹集资金41亿元。全年各项贷款余额508亿元，增长25.2%，存贷比达66.4%，较去年末提高8.1个百分点，增幅居全省前列。实施工业项目预审会审制度，提高投资强度，土地节约率达28%以上。
　　全面推行工程招投标有效最低价评审办法，深入实施“八分开”制度，累计审减、节约资金9.2亿元。科技创新能力进一步提升，高新技术产业总产值、增加值和专利申请量、授权量4项指标增速居全省首位。
　　（四）重招商搭载体，发展基础更加夯实招商引资成效显著。坚持市领导带头招商、小分队驻点招商，推进产业招商、以商招商、委托招商。完善招商引资“六个一”和招商项目预审机制，加大重大在谈项目及签约项目协调调度，全市引进投资规模5000万元以上新建项目105个，合同引资额330亿元，其中50个亿元以上项目合同引资额208.7亿元。105个项目中在建93个，竣工12个。投资49亿元的天裕高端铸造循环经济产业园落户淮北，投资30亿元的恒泰国际商城前期工作加快推进，国购城市综合体、美信铝业、温州食品工业园等一批重大招商项目开工建设。全市累计引进市外资金501.5亿元，增长33.4%，实际利用外资3.7亿美元，增长23%。
　　园区平台扩容升级。出台开发区转型升级指导意见，完善市开发区体制机制，六大开发区完成工业总产值712亿元，占全市工业比重达50.4%，实现税收24.5亿元。积极化解规划审批、土地征收、居民安置、建设资金等难题，市经济开发区新区一期20平方公里基本建成，成为淮北未来发展的新平台、新引擎、增长极。
　　县区完成园区基础设施投资11亿元，扩区10.7平方公里。濉芜现代产业园获省政府批准和重点支持，三年将投资20多亿元，打造15平方公里产业新城。临涣工业园获批全省唯一的国家级循环经济示范园区，杜集经济开发区成为省级矿山机械装备高新技术产业基地。总投资20亿元，占地2300亩，可容纳8万名师生的职教园区开工建设，成为推动淮北转型发展的人才支撑平台。
　　（五）抓建设重管理，城市功能不断完善城市建设加快推进。全年实施道路、管网、桥梁、绿化等城建重点项目43个，完成投资24.9亿元。淮海东路、沱河路等6条改造或新建道路具备通车条件。两年投资18.1亿元，改造提升老城区功能。完成多条街巷、小区改造，人民路等8条路巷铺设沥青罩面，改造升级5.1万平方米人行道，新建、改造港湾式公交站亭38座，完成宿丁路等17条道路绿化工程，新增绿地面积38.6万平方米。投入1亿元，铺设供水管网50余公里、天然气管网16.5公里，杜集、烈山污水处理厂开工建设。经过改造提升，道变坦，水变清，城变绿，城市面貌显著改观。
　　城市品质大幅提升。积极推进采煤塌陷区、矿山地质环境综合治理与生态恢复工程，投资1.4亿元，实施重点项目14个，治理土地6万亩，形成独具特色的湖光山色。投资5000万元，全面完成中小河流治理工程。“森林增长工程”正式启动。完成东部山场绿化1万余亩，绿色长廊建设工程70公里，获评“全国国土绿化突出贡献单位”。坚持征收与安置并重，出台《淮北市国有土地上房屋征收程序暂行办法》，开工建设安置房273万平方米。在2012年城市文明程度指数和未成年人思想道德建设工作测评中，我市名列全国地级提名城市第2名和地级城市第37名，为争创全国文明城市奠定了坚实基础。
　　（六）惠民生促和谐，社会建设统筹推进民生成果城乡共享。投入资金21亿元，全面完成34项民生工程，人均受益1000元。城乡最低生活保障标准不断提高，居民基本医疗保险和养老保险实现全覆盖。2.4万人参加新型农民和就业技能培训。2.8万名家庭困难学生得到资助。3所敬老院、4项农村清洁工程、9所乡镇公办幼儿园、27个校舍安全工程建成使用，1.2万套保障性住房基本建成，1300户农村危房、30座农村公路危桥完成改造，解决了10.8万农村居民饮水安全问题和2.1万户居民低电压整治问题。21个公共文化服务信息化建设项目、61个广播电视“村村通”工程、155个农家书屋投入使用。连续两年增设职业技能教育资助项目，我市职业院校2.1万名淮北籍在校生享受资助政策。积极响应人民群众的新要求，顺利完成1路、2路和18路个体中巴车公交化改造。12.8万名特殊群体获优惠乘坐公交车财政补贴。
　　社会事业全面进步。市财政用于社会事业投入73亿元，占财政总支出的72%，实现吃饭财政向民生财政转变。深入实施城乡居民收入倍增规划。扎实推进创业富民工程，我市被国务院授予“全国创业先进城市”。全年新增城镇就业7.69万人，新增农村劳动力转移就业1.42万人。学前教育快速发展，完成76所义务教育学校标准化建设，淮师大新校区一期建成使用，市委党校新校区主体封顶。新农合保障水平大幅提升，县级公立医院综合改革全面启动。隋唐大运河柳孜遗址二期发掘进展顺利。成功举办市九运会。人口和计划生育、外事侨务、民族宗教、保密、档案、目标管理、地方志工作不断加强，地震、气象、防灾减灾工作扎实推进，国防动员、双拥、优抚安置和人防工作取得新成绩，妇女、儿童、老龄、残疾人和红十字事业实现新发展。
　　社会大局和谐稳定。加强城乡社区建设，稳步推进社区综合管理体制改革，社区网格化管理基本实现全覆盖。全面实行居住证制度，流动人口服务管理切实加强。深入开展“领导干部大接访”活动，深化以群众工作统揽信访工作试点，妥善化解了一批信访积案，信访维稳工作连续4年受到省委、省政府表彰。价格监测工作获全国先进。治超工作走在全省前列。社会管理综合治理不断加强，安全生产形势总体平稳，食品药品监管体系逐步完善，群众安全感、幸福感指数保持皖北第一、全省领先。
　　（七）转作风提效能，政府自身建设进一步加强扎实开展“保持党的纯洁性、迎接党的十八大”主题教育实践活动。积极推进政府工作创新，金融、集约用地、职业技能教育、创业富民工程四项政府创新走在全省前列。深化纠风专项治理，积极开展“转作风、提效能、促转型”和民主考评“百名科长”活动，机关作风、行政效能、服务水平进一步提升。深入开展行政审批制度改革，清理规范涉企行政事业性收费，我市成为全省行政许可项目最少、审批流程最优的城市之一。
　　自觉接受人大法律监督、政协民主监督和社会舆论监督，加强与各民主党派、工商联、无党派人士协商，密切与工会、共青团、妇联等人民团体的联系，认真听取各方面意见和建议，全年办理省、市人大代表议案、建议93件，政协委员提案145件，满意度98%以上。办好政风行风热线和说办就办栏目，办理市长信箱、网上留言1942件，举办在线访谈16期，累计解决群众反映问题5471个。完善政府信息公开机制，健全财政预决算公开制度，实施公务卡制度改革，经济责任审计和廉政风险防控工作进一步加强。
　　各位代表！2012年各项任务的完成，为本届政府的任期目标划上了圆满句号。回顾过去五年，全市人民负重奋进、求真务实，有效应对金融危机冲击，战胜前进中遇到的各种困难和挑战，用激情与拼搏、辛勤与汗水，谱写了淮北加快转型、加速崛起、富民强市的新篇章。
　　五年来，我们致力跨越发展，综合实力大幅攀升。地区生产总值年均增长13.1%，连跨4个百亿元大关，总量实现翻番。人均生产总值由12738元增加到30100元，增长2.4倍。财政收入年均增长23.2%，总量增长2.8倍。五年累计完成固定资产投资1862亿元，年均增长30.4%，总量是上一个五年的4倍。销售收入超2000万元工业企业由109家增至620家，规模以上工业增加值年均增长17.2%，总量增长2.2倍，工业主导地位更加巩固。
　　五年来，我们致力调优结构，转型发展成效明显。非煤产业产值占全市工业总产值的比重由26.6%上升到65%。机械制造、食品工业产值分别增长27.3倍和7.
　　倍。县区生产总值、财政收入和规模工业增加值占全市比重分别提高13.2、5.8和22.5个百分点，成为全市转型发展的重要增长极。非公经济增加值五年增长1.6倍，年均增长20.7%。城乡环境质量达功能区要求，节能减排成效明显，资源综合利用率大幅提升。
　　五年来，我们致力夯实基础，潜力后劲不断增强。五年引进市外资金1495.7亿元，增长4.2倍，年均增长33.1%。资源枯竭城市转型优惠政策展期5年，将获得中央财政25亿元以上资金支持。
　　被列为全国首批工矿废弃地复垦利用试点市，未来三年可获2万亩土地利用指标。采煤沉陷区综合利用列为全省首批试点，已获国家财政3亿元扶持资金。淮北调水工程、南坪港开工建设。泗许高速淮北段建成通车，全市所有乡镇半小时内可通达高速。符夹线扩能改造项目列入铁道部改造计划，总投资近70亿元，三年后淮北将跨入“高铁时代”。
　　五年来，我们致力提升形象，城乡面貌深刻变化。城市建成区五年扩展23平方公里，城镇化率达56%。新增城市道路108公里，改扩建农村公路1231公里，城市管理和农村环境整治得到加强。
　　先后荣获全国创业先进城市、全国文明城市提名奖、全国科技进步先进市、全国无障碍建设先进城市和全省首届、第二届文明城市称号。五年来，我们致力促进和谐，社会建设全面发展。城镇居民人均可支配收入由11644元增加到20650元，农民人均纯收入由3374元增加到7480元，年均分别增长12.1%和17.2%。五年累计投入民生工程资金66亿元，人均受益3000元。累计新增城镇就业21.1万人，农村劳动力转移就业9.6万人。省级示范高中增至8所，淮北煤师院更名为淮北师范大学。市图书馆、文化馆、矿山博物馆、方志馆建成并免费向市民开放。我市成为国家知识产权试点城市。公共卫生服务体系实现城乡全覆盖。两次获评全国文化体制改革先进地区，连续两年入选中国最具幸福感城市百强榜单。
　　各位代表！五年的成绩鼓舞人心，取得的经验弥足珍贵，创新的实践影响深远。回顾总结五年奋斗历程，我们深切体会到，做好淮北的工作，必须坚持发展第一要务，必须坚持招商第一要事，必须坚持改革创新第一动力，必须坚持民生第一目标，必须坚持落实第一真功，强化责任、真抓实干，坚持为人民群众做好事办实事解难事，用实干实效创造更加幸福美好的明天。
　　各位代表！五年的开拓奋进，五年的辉煌成就，奠定了淮北科学发展、全面转型的坚实基础，开启了加快发展、富民强市的崭新局面。这是市委统揽全局、正确领导的结果，是市人大和市政协监督支持的结果，是全市人民同心同德、奋发有为的结果，是历届历任老同志打下坚实基础的结果。
　　在此，我代表市人民政府，向全市广大工人、农民、知识分子、干部，向驻淮解放军指战员、武警官兵和政法干警，向各民主党派、工商联、各人民团体，向关心、支持淮北发展的社会各界人士，向在我市创业的投资者、建设者，表示衷心的感谢并致以崇高的敬意！回顾过去，成绩固然令人欣慰，但总体来看，我市经济总量不大、发展速度不快、产业结构不优问题仍然突出；大项目少，规模企业少，煤炭资源枯竭，缺水缺地缺发展空间，发展后劲不足；投资环境还不够优，政府效能建设和绩效管理水平还不高，少数部门和政府工作人员精神状态不佳、工作作风不实，形式主义、官僚主义等现象依然存在，反腐倡廉建设力度需进一步加大。对此，我们将高度重视，并在今后的工作中认真加以解决。
　　****二、跨越赶超、加速崛起的美好前程****

    “十二五”规划提出，全面实施“2256”工程，争当皖北崛起排头兵，奋力跻身全省第一方阵。未来五年，是实现这一宏伟目标的决胜期，也是全面建成小康社会的关键期。纵观大势，发展的机遇和条件前所未有。我国仍处于大有作为的战略机遇期，发展长期向好的趋势没有变，为我市加快发展提供了长期稳定环境。中原经济区规划上升为国家战略，中央支持资源枯竭城市转型力度加大，省委、省政府支持皖北振兴政策更加普惠，我市迎来政策叠加的黄金发展期。淮北调水工程有效解决了我市发展长期缺水的难题，高铁时代的来临，为我市加速融入长三角、环渤海经济圈和中原经济区，提供了难得的历史机遇。随着工业化、信息化、城镇化和农业现代化加速推进，产业和消费加快升级，持续投入效应不断释放，加快发展的内生动力显著增强，加上我市工业化、城镇化基础较好的优势，我们完全有能力、有条件在新的起点上实现更优质量、更高效益、更快速度的发展。审视自身，面临的困难和挑战尤为严峻。国际金融危机影响深远，国内经济增长下行与物价上涨压力相互交织，发展的不确定、不稳定因素增多。当前煤炭市场低迷、煤价下跌，财政税收增幅下滑，充分暴露出淮北的“软肋”。
　　随着煤炭“十年黄金期”结束，我市加快发展、转型发展的任务尤为急迫。特别是区域竞争更趋激烈，我市与发达地区的差距进一步拉大，争先进位、加速崛起的任务更加繁重。
　　形势复杂多变，机遇稍纵即逝。抓住机遇，我们就能赢得更大跨越；错失良机，我们就会落后一个时代。必须进一步深化市情认识，全面把握机遇，沉着应对挑战，在创新创造中展现新作为，在苦干实干中谋求新突破，无愧责任使命、不负人民重托！今后五年政府工作的总体要求是：全面贯彻落实党的十八大精神，以邓小平理论、“三个代表”重要思想、科学发展观为指导，按照市第七次党代会的战略部署，以推动科学发展、建设美好淮北为主题，以加快城市转型为主线，以“皖北领先、全省争先”为总定位，坚定不移地推进大投入、大招商、大建设、大发展，促进工业化、信息化、城镇化和农业现代化协调发展，加快建成惠及全市人民更高水平的小康社会，奋力实现跨越崛起。
　　今后五年的奋斗目标是：
　　—— — 经济实力更加坚实。主要经济指标增幅超过全省平均水平，到2017年，实现地区生产总值、财政收入和固定资产投资比2012年翻一番，分别达到1300亿元、210亿元和1200亿元以上。
　　—— — 城乡发展更加协调。城市建成区面积扩至100平方公里、人口达到100万人，城镇化率达到61.5%。一批重点中心镇和特色小城镇加快崛起，美好乡村覆盖面达50%以上。
　　—— — 生态环境更加优美。强化节能减排，推进绿色、循环、低碳发展，水和大气环境质量显著提高，森林覆盖率达21.3%以上，天更蓝、山更绿、水更清，人居环境明显改善。
　　—— — 人民生活更加幸福。城镇居民人均可支配收入和农民人均纯收入分别突破4万元和1.45万元。社会保障更趋完善，基本公共服务均等化加快推进，人民群众幸福指数进一步提升。
　　—— — 社会发展更加文明。科教文卫事业全面发展，公民道德素质普遍提高。积极争创全国文明城市，不断提升社会管理水平。
　　围绕上述要求和奋斗目标，找准着力点，寻求突破口：
　　（一）加快产业转型升级，构筑现代产业体系。牢牢把握发展实体经济这一坚实基础，调整优化产业结构，推动产业向多元化、规模化方向发展，着力打造煤电与非煤电产业两个“千亿板块”，构建富有特色、更具竞争力的现代产业体系。延伸煤、提升煤、转化煤，推动煤炭资源精深加工，加快煤焦化、煤电材、煤焦电铸一体化发展，到2017年，力争煤电产业板块年产值突破1200亿元。推进战略性新兴产业和传统优势产业融合发展，到2017年，力争食品、机械制造、纺织服装和新型建材四大非煤产业板块年产值突破2000亿元；加快培育铝基新材料、生物医药、电子信息等若干百亿元以上新兴产业。打造皖北特色农产品加工基地，争创中国食品名城。加快发展文化旅游等现代服务业，打造区域性休闲旅游目的地。
　　（二）加快新型城镇化进程，促进城乡一体化发展。城镇化是扩大内需的最大潜力、经济增长的巨大引擎。坚持以规划为先导，以提升城镇综合承载能力为重点，优化城乡空间布局，着力构建“一城三镇三轴”的现代城镇体系。做大做强中心城区，提升我市在中原经济区地区性中心城市地位，加强县城和中心镇规划建设管理，全面推进美好乡村建设，着力打造“皖北江南、文明淮北”。坚持工业反哺农业、城市支持农村和多予少取放活方针,促进城乡要素平等交换、公共资源均衡配置，逐步实现城乡基本公共服务均等化。坚持“三化”同步、产城一体，推动开发园区转型升级，促进城乡产业互动发展。深化城乡一体化综合配套改革，着力在城乡规划、基础设施等方面推进一体化，引导人口向城镇集中、工业向园区集中、土地向适度规模集中。
　　（三）大力推动全民创业，加快富民进程。企业是发展之基，创业是富民之源。全面实施创业富民工程，以创业带动就业，以创业推动发展，以创业加快致富。着力营造“崇尚创业、鼓励干事、褒奖成功、包容失败”的创业环境，建立政府促进、社会支持、全民参与、充满活力的新型创业机制，形成百姓创家业、能人创企业、干部创事业的浓厚氛围。全面激活民间资本，积极鼓励、支持和引导非公经济加快发展，努力把淮北打造成为皖北乃至中原经济区最适宜创业的城市之一。
　　（四）推进创新驱动发展，构建区域创新体系。创新是推动发展的不竭动力。紧紧抓住增强自主创新能力这个中心环节，加快构建以企业为主体、市场为导向、产学研相结合的技术创新体系，努力建设创新型城市。建成一批企业技术中心和研发机构，提升自主创新能力，在若干领域形成具有自主知识产权的专利技术和名优品牌产品。坚持把人才作为第一资源，创新人才培养和引进模式，激发人才创造活力，使人尽其才，物尽其用，各种生产要素优化配置和集聚，成为创造财富的源泉。
　　（五）加强和谐社会建设，提高居民幸福水平。和谐是发展之本，幸福是群众所盼。顺应人民群众新期待，更加注重包容性增长和共享式发展，让人民群众得到更多实惠。更加注重发展社会事业、改善民生，使人民群众学有所教、劳有所得、病有所医、老有所养、住有所居。促进文化事业全面繁荣和文化产业跨越发展，增强文化软实力。坚定争创全国文明城市目标不动摇，按照“工作上求精、建设上求美、成效上求高”的总体要求，做足优势，突出特色，着力打造宜居、宜业、宜游的精致淮北。突出社会管理创新，引导和加强社会组织建设，增强城乡社区服务功能。建立健全安全生产和食品药品安全监管机制，加强社会治安综合治理，创造良好的社会环境。
　　****三、开拓进取、富民强市的崭新征程****

    今年是全面贯彻落实党的十八大精神的第一年，也是新一届政府的开局之年，做好今年各项工作意义重大。综合考虑，今年经济社会发展的主要预期目标是：地区生产总值增长12%；财政收入增长10%；规模以上工业增加值增长15%；固定资产投资增长25%；社会消费品零售总额增长15%；城镇居民人均可支配收入增长15%，农民人均纯收入增长16%；城镇登记失业率控制在4.1%以内；节能减排完成年度目标任务。
　　实现上述目标，重点抓好八个方面的工作：
　　（一）坚持工业强市战略不动摇，进一步强化工业经济的支撑作用。突出工业主导地位，树立企业为本理念，促进产业结构优化升级，着力提升工业经济增长的质量和效益。
　　扶持企业壮大规模。深入实施“万千百十”工程，以扶持成长性中小企业、壮大骨干企业为重点，加快市场主体培育，鼓励更多大企业做大做强，更多小企业做专做精，力争全年新增规模企业100户，销售收入亿元以上企业60户、5亿元以上企业5户。强化经济运行调度，完善落实扶持企业发展的政策措施，切实减轻企业负担，促进企业效益持续好转。
　　加强与央企、省属大企业对接，深化与淮矿集团、皖北煤电、中煤矿山集团和淮海实业战略合作，进一步发挥大企业对我市经济发展的支撑和带动作用。
　　加快发展优势产业。做大煤电化主导产业，实施煤炭产能接续和产业升级改造工程，稳定市域内煤炭产量，扩大电力装机容量，支持临涣煤焦化电延长产业链条，实现综合利用、绿色发展。实施“百亿口子”行动计划，发挥口子酒业发展潜能。延长食品、机械制造产业链，大力发展铝基型材、节能墙材等新型建材，引导纺织服装产业精细化、品牌化、园区化发展。充分发挥新兴产业引导资金和城市转型创投基金作用，重点发展新能源、新材料、生物医药等战略性新兴产业，力争新兴产业实现产值270亿元，增长25%以上。
　　提高自主创新能力。要素推动资源有限，创新驱动潜力无限。
　　充分发挥科技创新引领带动作用，积极开展“科技提升园区和产学研合作推进年”活动，扎实推进国家技术创新工程试点工作，推动科技、经济融合发展。加快市科技孵化器建设，加大紧缺高层次人才引进力度，积极引进科技创新领军人才和研发团队。加强技术创新研发和产学研合作平台建设，加快培育创新型产业，力争全年新增国家级高新技术企业8家，省级高新技术产品50个，高新技术产业产值突破300亿元，确保蝉联全国科技进步先进市。
　　（二）加快城乡统筹步伐，构建城乡一体化新格局。坚持以城带乡、以工促农，在加快新型城镇化进程中同步推进农业现代化、城乡一体化发展。
　　促进城乡融合发展。编制完成《淮北市城市发展战略规划》，积极推进城市东进西延南扩北拓，重点做好东部新城核心区规划布局和北部新城规划，全面拉开城市框架。加快临涣、渠沟、烈山等中心镇建设，着力培育一批生态宜居、功能完善的特色城镇。
　　加快户籍制度改革，有序推进农业转移人口市民化。推进城镇基础设施向农村延伸、公共服务向农村覆盖，促进城乡共建共享、一体发展。开工建设S235濉唐路（宋疃—南坪段）、G3京台高速连接线(古饶—石台段)，续建S202萧淮路改建工程，升级改造农村公路76公里。力争S101合相路改建工程开工建设。
　　大力发展现代农业。继续推进小麦高产攻关活动和玉米振兴计划，稳定粮食生产。推进和规范农村土地承包经营权流转，力争新增土地流转面积10万亩。创新农业经营体制机制，提高农业组织化、规模化程度，重点扶持100家农业产业化龙头企业，创建100个标准化规模种养小区，力争培育2个产值分别超50亿元和100亿元的农业产业化示范区。加快百善、渠沟、塔山、高岳4个现代农业示范区和五铺、古饶等循环农业示范园建设，重点培育10个“一村一品”示范村。投资2.1亿元，扎实开展农田水利基本建设，新增和改善灌溉面积29.2万亩、除涝面积30.5万亩。
　　加快美好乡村建设。认真落实建设美好乡村战略部署，科学编制村庄建设规划，合理确定村庄布点，用好1.亿元市级美好乡村建设专项资金，重点支持南山、华家湖、张集、蒙村等首批37个中心村基础设施和公共服务配套，力争美好乡村建设走在全省前列。
　　做大做强县区经济。赋予县区更多的管理权限，落实扩权强镇政策。加快发展机电制造、农副产品加工、矿山装备、新材料等特色优势产业，壮大县区工业体量，进一步提升县区经济在全市经济中的比重。完善科学发展分类考核办法，激活县区发展潜能，支持三区争先进位，支持濉溪县争创全省十强。
　　（三）推动服务业提速增效，促进服务业跨越发展。完善政策措施，优化城乡消费环境，促进服务业发展提速、水平提升，力争全年新增限额以上商贸类企业100家、限额以上其它类服务业单位100家。
　　改造提升商贸等生活性服务业。提档升级老城区商贸流通、住宿餐饮等传统服务业，推进淮海路、孟山路核心商圈资源整合、业态完善和品牌提升。统筹谋划东部新城区商业布局，完善商业服务功能。推进特色商业街、城市综合体建设，加快国购广场、乐天玛特、中泰国际广场、红星美凯龙、国购汽车文化园、恒泰国际商城等一批重点项目建设。积极发展家政、养老等社区服务业。支持和促进房地产市场健康稳定发展。
　　继续推进“万村千乡”市场工程、“新网工程”和“农超对接”，进一步激活农村消费。
　　加快发展物流等生产性服务业。围绕促进工业转型升级和加快农业现代化进程，大力发展现代物流业，加快推进凤凰山、百善、段园等七大物流园区建设，支持翔宇物流建成皖北最大的物流基地，重点扶持发展淮矿物流园、百路、蓝宇等第三方物流。大力发展职业技术教育，树立特色品牌。
　　规范发展法律服务、会计审计、资产评估等专业商务服务。
　　大力发展文化旅游等现代服务业。促进文化创意、金融保险、健康养生、商务会展、设计研发等新兴业态加快发展。积极开展“旅游发展突破年”活动，推动旅游业大跨越，力争实现接待游客、旅游收入分别增长31.4%和32%。促进文化旅游深度融合，扎实推进口子文化博览园、洪庄文化产业园、儿童梦幻王国、龙脊山风景区开发建设。高水准规划建设“四季榴园”风景区和石榴博览馆，力争年内成功创建国家4A级景区。
　　积极开展星级农家乐创建活动，提高经营服务档次。
　　（四）加快推进项目建设，持续增强发展后劲。坚持以扩大投资需求、增加有效投入稳增长、促转型，充分发挥投资对经济增长的拉动作用。
　　狠抓项目实施。完善市领导督查重大项目工作机制，年内新建、续建重点项目336个，完成投资426亿元。开工建设天裕70万吨高端铸造、平山电厂等一批重大项目，确保大唐虎山电厂和铚城、中泰、铁佛变电站年内竣工投产，金龙电子、美信铝业年内建成，口子工业园、国购汽车文化园、天正科技主体完工。加快宝迪总部、温州工业园、淮北调水、浍河复航和南坪港续建工程建设，启动南坪船闸建设工程。积极推进职教园区建设，力争一期工程今年秋季建成使用。

    积极做好项目谋划和前期工作。落实重大项目谋划工作机制,着力谋划、储备一批打基础、管长远、增后劲的大项目,全年谋划5亿元以上重大项目50个。重点围绕“三农”、保障性住房、基础设施建设、节能减排和生态建设、战略性新兴产业以及民生工程、社会事业等方面,加大项目谋划、编报和争取力度,积极争取中央和省级项目资金。加快推进符夹线扩能改造等项目前期工作,力争年内开工建设。
　　(五)强力推进招商引资,不断提升园区承载能力。始终坚持招商引资第一要事不动摇,坚定不移抓招商、搭载体,推进大招商、招大商,实现借力发展,助推城市转型。
　　加大招商引资力度。推动招商引资工作再升温、再提速、再加压,确保年内引进固定资产投资规模亿元以上项目100个。完善招商引资考核奖惩办法,深化招商引资机制体制创新,优化“六个一”工作机制,全面激发招商潜能。扩大食博会、煤博会品牌效应,积极组织招商推介活动。立足招大引强,把主攻点放在世界500强、国内500强和上市公司,着力引进一批投资体量大、科技含量高、产业关联度强的大项目、好项目。进一步强化载体招商功能,加强市开发区专业招商队伍建设,三区一县和市开发区至少各引进一个5亿元以上的项目。严格项目预评估制度,强化合同执行,切实提升选资质量。
　　推进园区加快建设。完善各类开发区规划,优化产业和空间布局,推动开发区转型升级。加快园区基础设施建设,提升产业集中度和投资强度,力争六大开发区年内扩区7.4平方公里。完善市开发区体制机制,推进市开发区新区与龙湖工业园整合,加快市开发区新区二期建设,确保运河路等8条道路和梧桐大桥建成通车,创业园及59万平方米安置房交付使用,20平方公里规划范围内全面具备企业入驻条件。加快濉芜现代产业园建设,支持市、县开发区申报国家级开发区,支持临涣工业园全面推进国家级循环经济示范园区建设,杜集、凤凰山经济开发区分别争创国家级矿山机械装备高新技术产业基地、食品工业科技园,确保龙湖工业园成功创建省级高新技术开发区。
　　(六)深化改革推动创新,激活发展的内生动力。把改革创新作为推动科学发展的先导,在深入学习十八大精神中激发全市人民创新创造活力,充分释放“改革红利”,形成加速崛起的强大合力。
　　继续深化各项改革。稳步推进事业单位分类改革。积极做好县级公立医院综合改革。深化财税体制改革,优化财政支出结构。完善城市建设管理“八分开”运行体制,探索土地收储融资平台改革,成立市保障房建设投资公司。继续抓好工矿废弃地复垦利用试点,大力推进农村土地整治,积极争取增减挂钩周转指标,力争全年新增建设用地指标8000亩。
　　持续推进金融创新。濉溪县信用联社完成改制,推动中国银行、浙江泰隆银行等在淮设立村镇银行。推广重点中小企业助保金贷款、农村土地承包经营权抵押贷款等创新业务,探索集体建设用地使用权、林权等抵押贷款。支持淮矿集团组建财务公 司,支持淮矿股份、口子集团年内上市,加快巨成化工等后备企业上市步伐,积极探索中小企业私募债券试点工作。发挥小额贷款公司、担保公司作用,重点面向农户及小微企业提供资金服务。促进政银企对接,力争全年新增贷款100亿元,增长20%以上。
　　不断扩大对外开放。加快淮北发展,必须拓宽对外开放的广度和深度。既要大力“引进来”,招商引资、招才引智,又要鼓励企业“走出去”,利用两个市场,优化资源配置。加强对外经贸交流与合作,帮助企业争取国家政策性银行优惠贷款和出口风险担保,努力保持出口稳定增长。深化与长三角区域、中原经济区、淮海经济区城市之间协作。发挥段园工业集中区“飞地”优势,积极主动接受徐州的辐射和带动。
　　大力发展非公经济。把加快非公经济发展提升到淮北可持续发展的战略高度来认识和推动,大力推进全民创业,努力营造创业光荣、创业有功的社会氛围,让资本活力迸发,创新智慧涌流。创优发展环境,对非公经济一视同仁,鼓励外来民营企业来淮投资兴业,支持本土民营资本做大做强,深入推进与全国知名民企合作发展。认真贯彻国家促进非公经济发展“新36条”,落实完善减负惠企政策措施,强化对小微企业扶持。实施非公经济倍增计划,力争全年新增私营企业1600户、个体工商户1.5万户,非公经济增加值占全市生产总值比重提高到42%以上。
　　(七)加快生态宜居城市建设,提升城市功能品位。在不断拉开城市框架、扩大城市规模的同时,更加注重改善生态环境,提高人居质量,着力塑造“皖北江南、文明淮北”城市形象。
　　持续完善城市功能。计划投资9.2亿元,实施城市畅通工程,确保淮海东路、梧桐南路、沱河路、东山路北段全线贯通,完成相山南路等15条道路综合改造提升工程。投资3000万元,推进老旧小区综合整治。改造资源型城市及独立工矿棚户区120万平方米,竣工50万平方米。建成安置房240万平方米。加快推进淮师大新校区二期前期工作,启动建设市政务新区、老年活动中心。
　　加强生态城市建设。抓好城区东部山场绿化和森林增长工程,确保完成1.6万亩城区荒山绿化和7.3万亩年度造林任务,争创省级森林城市。综合治理城市水系及水环境,扎实推进全省首批采煤沉陷区综合利用试点工作,加快建设南湖、中湖、东湖国家湿地公园。推进市、县区污水处理厂与配套管网工程建设,支持有条件的乡镇加快污水设施建设,进一步提高城乡污水处理率。强化饮用水源保护,依法取缔保护区内违法建设项目和活动,确保人民群众喝上放心水。
　　深入推进文明城市创建。进一步完善文明创建长效机制,推动文明城市创建活动向镇村延伸、向深层次拓展。加强市民文明素养教育,大力弘扬“好人”文化。创新城市管理体制机制,在相山区试点实施考评奖惩机制,推进管理重心下移和环卫作业市场化,加大督查考核奖惩力度,构建市级管总、县区实施、监管分离的城市管理工作新格局。全面推行社区网格化管理,提升社区公共服务水平。持续开展“清洁乡村、美化家园”活动,积极整治城市综合环境。
　　(八)加强以改善民生为重点的社会建设,提升群众幸福感和满意度。社会建设是中国特色社会主义事业“五位一体”总布局中的重要内容,事关人民群众幸福安康,事关社会大局和谐稳定。
　　巩固提升民生工程。进一步加大财政投入力度,实施34项民生工程。实施建设美好乡村公共服务体系奖补、生活无着人员社会救助、基本公共卫生服务和农村文化建设等专项补助。农村低保人均补差水平和重度贫困残疾人救助标准提高10%,扩大经济困难学生资助范围。提高新农合、城镇居民医疗保险财政补助标准,扩大社会保险覆盖面。加快城乡养老服务体系建设,继续实施城乡居民养老保险、计划生育家庭奖扶、城市低收入家庭住房保障政策。
　　统筹发展各项社会事业。全面落实收入倍增规划,提高城乡居民收入。实施更加积极的就业政策,重点抓好高校毕业生、城镇就业困难人员和失地农民就业,新增城镇就业3.5万人、农村劳动力转移就业1.2万人。深入推进创业富民工程,完成就业技能培训1万人以上。全面完成学前教育三年行动计划,促进义务教育均衡发展。投资6000万元,完成63所义务教育阶段学校标准化建设。投入2000万元,实施教育信息化建设工程。继续推进职业教育超常规发展战略,争创1个国家级社区教育实验区。建成、启用市医疗紧急救援指挥中心。
　　扎实做好第三次全国经济普查工作。实施隋唐大运河柳孜遗址保护和展示工程,推进石山孜遗址文化公园建设,认真做好榴园村申报全国历史文化名村前期工作。发展群众体育事业,促进全民健身。统筹做好人口和计划生育工作。加快城乡公交一体化建设。加强价格监测预警,保持物价总水平基本稳定。
　　全力维护社会稳定。建立健全重大决策风险评估机制,从源头上避免和减少损害群众利益现象的发生。坚持以群众工作统揽信访工作,积极稳妥化解矛盾纠纷。强化社会管理创新,加快社区综合管理体制改革。全面实行居住证制度。深入实施“六五”普法工作。扎实推进“平安淮北”建设,健全社会治安防控体系,严厉打击违法犯罪。整治和规范食品药品市场秩序,保障人民群众饮食用药安全,建成启用食品安全网格化监管信息系统。严格落实安全生产责任制,积极开展“打非治违”专项活动,有效遏制重特大事故发生。推进军民融合式发展,确保实现全省双拥模范城“四连冠”目标。
　　各位代表!新形势、新任务,对政府工作提出了新的更高要求。“空谈误国,实干兴邦”。我们必须进一步加强政府自身建设,把实干作为第一本领,落实作为第一真功,朝夕勤勉,永不懈怠,始终做到为民务实清廉,奋力开创政府工作新局面。
　　一是奋发有为促发展。抓发展是政府工作第一要务,必须坚持不动摇、不懈怠、不折腾。不管面对多大困难,始终保持蓬勃朝气和昂扬锐气,敢于改革创新破难题,转型攻坚打硬仗。各级政府及工作人员要紧盯目标抓发展、突出实效抓发展,真正做到以实绩论英雄,以发展论成败。
　　二是以人为本办实事。牢固树立群众利益无小事的思想,深入基层,深入群众,把更多的时间和精力用在帮助群众解决实际 困难和问题上;珍惜民力,尊重民意,把有限的财力用到经济建设和改善民生上。坚持问政、取智、求计于民,善于从群众中汲取智慧和力量,切实办好顺民意、解民忧、惠民生的实事好事。
　　三是转变作风提效能。严格执行中央“八项规定”和省市具体要求,大力改进会风文风,开短会、讲短话,少发文、发短文。巩固“转作风、提效能、促转型”主题活动成果,加强督查考核,严格问责问效,坚决整治庸懒浮散。
　　健全民主评议机关制度,扎实推进政府绩效管理,着力整治不作为、不出力、不落实等问题,切实提高行政效能,全力优化发展环境。
　　四是务实清廉重实干。坚持依法行政,规范政府行为,切实做到公正文明执法。自觉接受人大及其常委会法律监督和工作监督,接受政协民主监督,虚心听取各民主党派、工商联、无党派人士意见。深化政务公开,加强政务服务体系建设。坚定不移反腐倡廉,建设清廉政府。严格落实党风廉政建设责任制,建立健全对领导干部、人财物管理使用、关键岗位的监督和制约机制,全面推行廉政风险防控工作,努力从源头上预防和治理腐败。加强征地拆迁、重点工程、政府采购等重点领域审计监督,严肃查处各类违法违纪案件。
　　各位代表!伟大事业源于伟大梦想。党的十八大为我们描绘出中华民族伟大复兴的强国之梦。让我们在中共淮北市委的坚强领导下,解放思想,与时俱进,开拓进取,求真务实,为夺取全面建成小康社会的新胜利，为建设文明富裕美丽幸福的新淮北而努力、而拼搏、而奋斗！
